# Biosorption of Fe(II) and Mn(II) Ions from Aqueous Solution by Rice Husk Ash

**DOI:** 10.1155/2014/973095

**Published:** 2014-06-01

**Authors:** Ying Zhang, Jiaying Zhao, Zhao Jiang, Dexin Shan, Yan Lu

**Affiliations:** School of Resource and Environment, Northeast Agricultural University, Harbin 150030, China

## Abstract

Rice husk ash (RHA), an agricultural waste, was used as biosorbent for the removal of Iron(II) and Manganese(II) ions from aqueous solutions. The structural and morphological characteristics of RHA and its elemental compositions before and after adsorption of Fe(II) and Mn(II) were determined by scanning electron microscopic (SEM) and X-ray fluorescence (XRF) analyses. Batch experiments were carried out to determine the influence of initial pH, contact time, adsorbent dosage, and initial concentration on the removal of Fe(II) and Mn(II) ions. Langmuir, Freundlich, and Dubinin-Radushkevich (D-R) models were applied to describe the biosorption isotherm of the metal ions by RHA. The correlation coefficient (*R* ^2^) of Langmuir and Freundlich isotherm models equals 0.995 and 0.901 for Fe(II), 0.9862 and 0.8924 for Mn(II), respectively, so the Langmuir model fitted the equilibrium data better than the Freundlich isotherm model. The mean free energy values evaluated from the D-R model indicated that the biosorption of Fe(II) and Mn(II) onto RHA was physical in nature. Experimental data also showed that the biosorption processes of both metal ions complied with the pseudo-second-order kinetics.

## 1. Introduction

Currently, the removal of heavy metal contaminants from aqueous wastewater is one of the most important environmental issues being researched. Once metal ions enter the environment, their chemical form largely determines their potential toxicity [1]. Besides the existence in aquatic ecosystem may cause harmful effects to organisms living in water and heavy metals also accumulate throughout the food chain and may affect the health of human beings [2–4].

Iron and manganese are found in groundwater and present in the form of Fe(II) and Mn(II) ions [5]. Fe(II) and Mn(II) often occur together in groundwater, but the concentration of manganese is found to be usually much lower than the concentration of iron [6]. Water percolating through soil and rock can dissolve minerals containing iron and manganese and hold them in solution [7]. Polluted water may cause taste, odor, color, or turbidity problems. Iron and manganese present in groundwater will cause a severe colour condition. When exposed to air, iron and manganese present in the water body become indissoluble and leave the water with brown-red colour. The problems caused by iron and manganese are not only aesthetic problems, but also indirect health concerns and economic problems [8]. Iron and manganese are apparent in drinking water supplies, especially iron. There are secondary standards set to constrain the emissions of iron and manganese ions. The secondary standard maximum contaminant levels (MCLs) for iron and manganese are 0.3 mg/L and 0.1 mg/L, respectively [9]. Therefore, it becomes necessary to remove these heavy metals from wastewaters by an appropriate treatment technology before releasing them into the environment [10].

A lot of methods are used to remove heavy metals because of the new and effective separation technologies. The most widely used methods for removing heavy metals from wastewaters include ion exchange [11], chemical precipitation [12], preconcentration [13], reverse osmosis [14], membrane filtration [15], and adsorption biological treatment [16–18]. Most of these methods suffer from some disadvantages such as high operational cost and are not suitable for small-scale industries or do not lead to a satisfactory result. Among these technologies, adsorption is a most common technique for the removal of heavy metal. This process seems to be more user friendly and effective if combined with appropriate bioadsorbent and regeneration steps. Activated carbon has been widely applied for removing heavy metals from water and wastewater [19–21]. Recently, the number of researches focused on the use of activated carbon as adsorbents is reducing due to their high capital and operational costs. Therefore, more interests have recently arisen in the investigation of low-cost adsorbents with a good sorption capacity to remove heavy metal ions from wastewater. For the past few decades, more researchers have concentrated on the use of agricultural wastes as adsorbents. Agricultural wastes such as fly ash [22], natural zeolite [23], wheat bran [24], bark and sawdust [25], peanut shells [26], and rice husk [27] have been developed for heavy metals removal from aqueous solution.

Rice husk is a kind of byproduct obtained from the rice mills and usually available in a large quantity of production [28]. Rice husk is mostly used as a fuel and burned in the boiler of various industries to produce steam, thus, conserving both energy and resources. The ash generated after burning the rice husk in the boiler is called rice husk ash (RHA). The RHA was collected from the particulate collection equipment attached upstream to the stack of rice-fired boilers. Since RHA is safe and available in plenty, and it has the possibility to function as an adsorbent, the objective of this work was to examine the adsorption characteristics of RHA to adsorb Fe(II) and Mn(II) ions from an aqueous solution. The effects of sorption parameters such as pH, contact time, adsorbent dosage, and initial concentration were examined. The equilibrium data were analyzed using Langmuir and Freundlich isotherm models. Kinetic studies were carried out, and the data were analyzed using pseudo-first-order and pseudo-second-order equations.

## 2. Experimental

### 2.1. Adsorbent Preparation of Highly Active RHA

The RHA was used as biosorbent for the biosorption of Fe(II) and Mn(II) ions. Samples of the biomass were collected from Heilongjiang Province, Shangzhi, surrounding rice mill. The sample was dried in an oven at 60°C for 3 hours, then put in dryer, and stored for later use.

### 2.2. Batch Adsorption Studies

Biosorption experiments were carried out by shaking 150 mL flasks containing 50 mL of Fe(II) and Mn(II) solutions of the desired concentration on a shaker machine at a revolving speed of 130 rpm, at 25°C. The mixture was filtered using an acid-cleaned 0.45 *μ*m Millipore filter and the concentration of Fe(II) and Mn(II) in the filtrate was determined by atomic absorption spectrometry (Model AA6800, Shimadzu, Japan). Effect of pH on the adsorption of Fe(II) and Mn(II) by RHA was studied from 1.0 to 8.0. The pH was adjusted with 0.1 mol/L HCl and NaOH solutions; a pH meter (pHS-3C, China) was employed for measuring pH values in the aqueous phase. The effect of contact time was studied by taking out the samples from the shaker at regular time intervals till equilibrium was reached. The effect of adsorbent dose was studied with different adsorbent doses ranging from 0.2 to 1.5 g/50 mL. The effect of initial concentration was studied from 5 mg/L to 40 mg/L, and initial solutions with different concentrations of Fe(II) and Mn(II) were prepared by proper dilution from stock of 1000 mg/L Fe(II) and Mn(II) standards. All experiments were repeated three times, and results presented are consequently the averaged values of replicate tests.

The X-ray fluorescence (XRF) spectrum analysis (Model Axios PW4400, PANalytical) was conducted for the element composition of raw rice husk ash. The surface morphology of rice husk ash was carried out by using a scanning electron microscope (Model S-3400N, HITACHI).

Sorption isotherms were conducted at sorbent dose of 0.5 g and varying the concentration of Fe(II) and Mn(II) from 2 mg/L to 40 mg/L in 150 mL flasks containing 50 mL Fe(II) and Mn(II) solutions. The pH was adjusted to 5.0 and 6.0, respectively. The mixtures were shaken in an oscillator at 130 rpm for 3 hours and at constant temperature.

The kinetic experiments were performed in continuously stirred flask containing 50 mL Fe(II) and Mn(II) solutions at concentrations of 20 mg/L from 5 min to 90 min at sorbent dose of 1 g and pH 5 and 6, respectively. Likewise, the mixtures were shaken in an oscillator at 130 rpm and at constant temperature. After filtering the mixture, the concentration of Fe(II) and Mn(II) in the filtrate was determined by atomic absorption spectrometry.

The percentage removal of Fe(II) and Mn(II) ions and equilibrium adsorption amount of Fe(II) and Mn(II) *q*_*e*_ (mg/g) were calculated by using the following equations:

(1) The  percentage  removal  of  Fe(II)  and  Mn(II)  ions =100(C0−Ce)C0.

Adsorption amount of Fe(II) and Mn(II) per gram (g) of adsorbent (mg/g) is

(2) $$\begin{array}{c} q_{e}=\frac{\left(C_{0}-C_{e}\right)V}{W}, \end{array}$$

where *C*_0_ is the initial concentration of Fe(II) and Mn(II) (mg/L), *C*_*e*_ is the equilibrium concentration of Fe(II) and Mn(II) (mg/L), *V* is the volume of the solution (L), and *W* is the mass of the adsorbent (g).

## 3. Results and Discussion

### 3.1. Characterization of RHA

In comparing the results of Fe(II) and Mn(II) adsorption based on percentages changes, before and after the addition of adsorbent, it can reflect the qualitative transformation and migration mechanisms of the elements and speculate the adsorption mechanism of Fe(II) and Mn(II) by RHA. XRF analysis on RHA before and after adsorption of Fe(II) and Mn(II) is shown in Table 1. After the RHA adsorbed Fe(II) and Mn(II), elements of Na disappeared, and elements of Mg, Al, K, and Ca decreased significantly. The adsorption process produced some sort of damage on the cytoderm of RHA, leading to the dissolution of intracellular substances; this resulted in ion exchange between Fe(II) and Mn(II) ions. Elements of Cl changed differently in adsorption Fe(II) and Mn(II). It indicated that Cl has produced different effects on Fe(II) and Mn(II) adsorption mechanisms. After adsorption of Fe(II) and Mn(II) by RHA, the content of Fe(II) and Mn(II) in the RHA increased from 0.175% and 0.183% to 0.293%, respectively. This result proved that the use of RHA to adsorb Fe(II) and Mn(II) is feasible.

**Figure 1:**
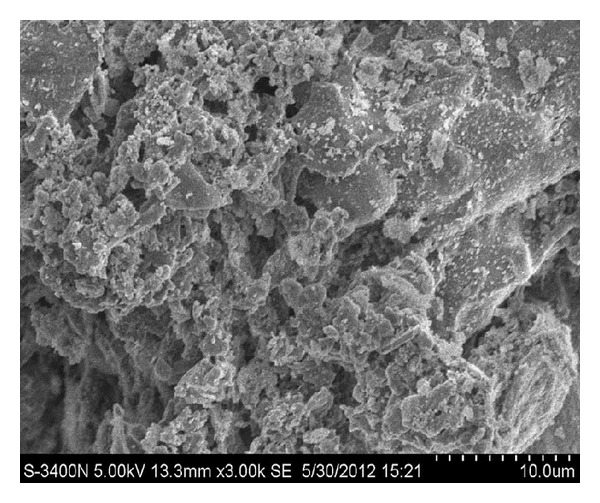
Scanning electron micrograph of RHA.

RHA is a porous material of Trass volcanic ash; the main ingredient is amorphous SiO_2_, up to 60%–97% content. Scanning electron micrographs of RHA are shown in Figure 1. The surface of RHA is porous, and the surface honeycomb holes can reach micron scale, about 10 microns. The internal structure possesses a number of irregular pieces of layered structure and reticulates. The cross section in the figure shows the irregular holes distribution within the RHA; this may be a result of the combined action of rice husk residue and RHA. The porous structure of RHA has a relatively large specific surface area, and this morphological property is conducive to the uptake of metal ions.

### 3.2. Effect of pH

**Figure 2:**
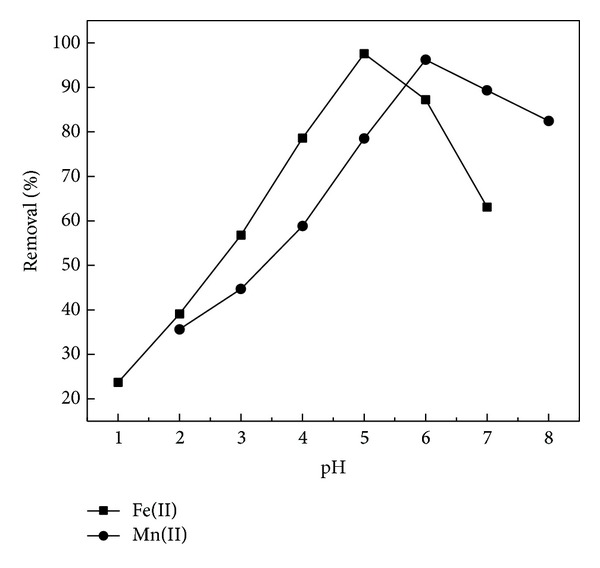
Effect of pH on adsorption of Fe(II) and Mn(II) by RHA (metal concentration: 20 mg/L; adsorbent dosage: 0.6 g/100 mL.).

pH is one of the most important factors affecting biosorption of metal ions. Differences in initial pH directly affect the competitive ability of hydrogen ions with metal ions for the active sites on the biosorbent surface [29]. The effect of pH on the biosorption of Fe(II) and Mn(II) ions onto RHA was studied at pH 1–8 and the results are presented in Figure 2.

It was observed that the removal amount was increased from 24 to 79% for Fe(II) ions and from 36 to 78% for Mn(II) ions, as pH was increased from 1 to 4 and 2 to 5, respectively. The maximum removal was found to be 98% for Fe(II) and 96% for Mn(II) ions at pH 5 and 6, respectively. This phenomenon partly attributed to the fact that when the pH values increased, biosorbent surfaces were more negatively charged and attracted metal ions with positive charges, thus causing the absorption onto the biosorbent surface [30]. But biosorption efficiency decreased after attaining the maximum biosorption limit. This could be due to the formation of soluble hydroxylated complexes of the metal ions and their ionized nature. Moreover, at higher pH levels, Fe(II) and Mn(II) would be converted into their hydroxide forms and get precipitated. So it could not be concluded that the removal of Fe(II) and Mn(II) was due to adsorption or due to precipitation. Above all the following experiments were carried out with pH values of 5 and 6, respectively.

### 3.3. Effects of Contact Time

**Figure 3:**
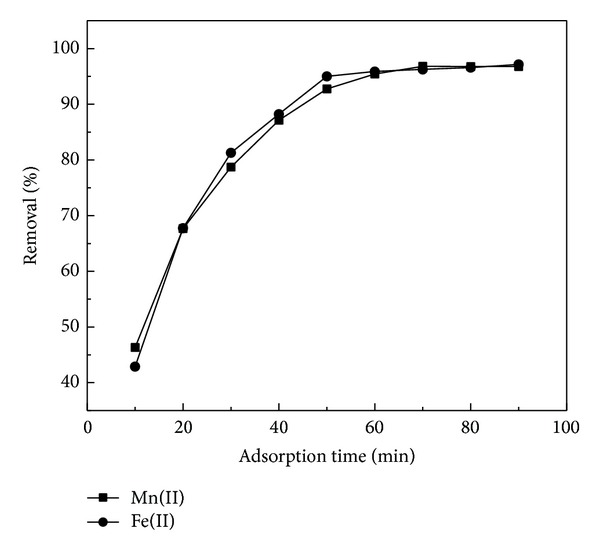
Effect of contact time on adsorption of Fe(II) and Mn(II) by RHA (metal concentration: 20 mg/L; adsorbent dosage: 0.6 g/100 mL; pH: 5 and 6, resp.).

The reaction time is one of the important factors that influence the adsorption process of heavy metals in a medium [31]. Selection of proper adsorption time of heavy metals in wastewater treatment has certain economic benefits. The effect of contact time on the uptake of Fe(II) and Mn(II) ions onto RHA was studied and is shown in Figure 3. It was observed that the percentage removal of Fe(II) and Mn(II) increased with increase in contact time up to 60 min. After this time there was no considerable increase. After 60 min, the biosorption efficiency for Fe(II) and Mn(II) was 96% and 95%, respectively, and 97% and 96% after 90 min, respectively. Therefore, the optimum contact time was selected as 60 min for further experiments. This result may be due to the use of vacant adsorption sites on the adsorbent surface. During the initial stage of sorption, a large number of vacant surface sites were available for adsorption. After a lapse in time, the remaining vacant surface sites were occupied due to repulsive forces between the solute molecules on the adsorbent surface and the bulk phase [32].

### 3.4. Effect of Adsorbent Dose

**Figure 4:**
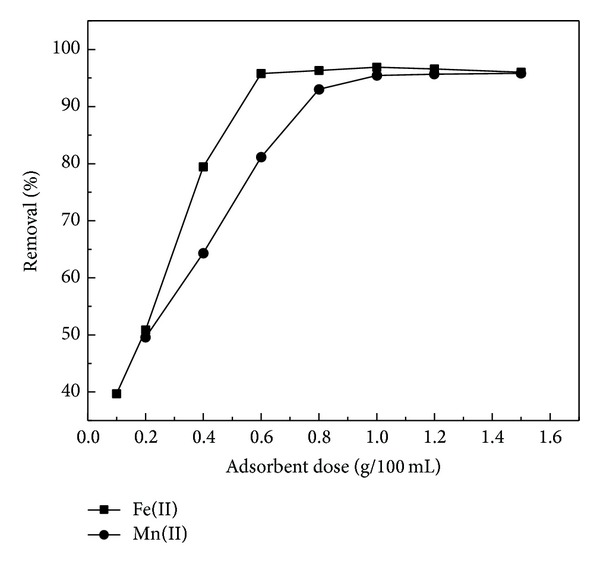
Effect of adsorbent dose on the adsorption of Fe(II) and Mn(II) by RHA (metal concentration: 20 mg/L; contact time: 60 min; pH: 5 and 6, resp.).

The study on the effect of adsorbent dose is necessary and very useful in order to find out the optimum amount of RHA required for the removal of Fe(II) and Mn(II) ions.  Figure 4 shows the effect of the adsorbent dose on biosorption of Fe(II) and Mn(II) ions. The biosorption efficiency of Fe(II) and Mn(II) was found to increase exponentially with the increase of adsorbent dose up to 6 and 10 g/L, respectively. This may be due to the increase in availability of surface active sites resulting from the increased dose of adsorbent. At maximum biosorption, 96% for Fe(II) and 95% for Mn(II), and at higher dosages, 12 and 15 g/L, biosorption was almost the same. This result can be explained as when the adsorption dose reached a certain rate, the adsorption site was used up, hence with reduced tendency of the particles to absorb any more ions to its surface, so removal rate of heavy metal ions no longer increased [33].

### 3.5. Effect of Fe(II) and Mn(II) Ions Concentrations

**Figure 5:**
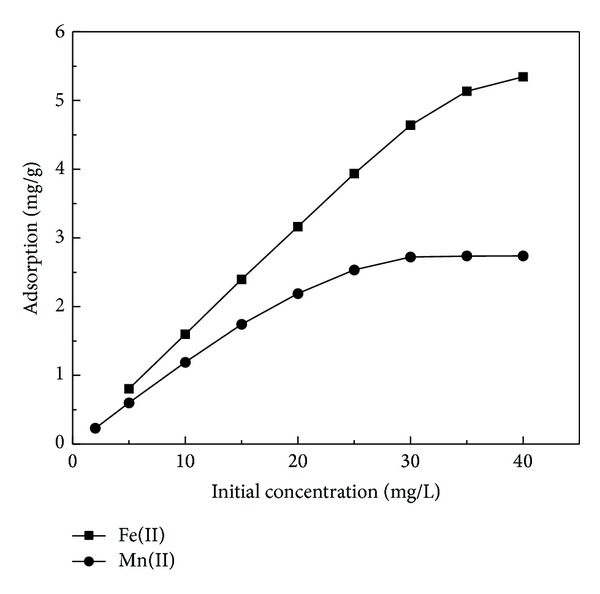
Effect of initial concentration on the Fe(II) and Mn(II) adsorption (adsorbent dosage: 0.6 g/100 mL; contact time: 60 min; pH: 5 and 6, resp.).

The effect of initial concentrations on the removal of Fe(II) and Mn(II) ions by RHA was studied and the result is given in Figure 5. The percentage removal was found to decrease exponentially with the increase in initial concentration of Fe(II) and Mn(II). As initial concentration of Fe(II) and Mn(II) increased from 5 to 40 mg/L, percentage removal decreased from 96% to 80% and 90% to 55%, respectively. This may be due to the lack of available active sites required for the high initial concentration of Fe(II) and Mn(II). Similar results have been reported in previous studies [34, 35].

### 3.6. Adsorption Kinetics

Adsorption kinetics, which is one of the important characteristics defining the adsorption efficiency of the surface of the adsorbent, describes the solute uptake rate. The kinetics of Fe(II) and Mn(II) adsorption was evaluated by applying two common models: (1) the pseudo-first-order kinetic model [36] and (2) the pseudo-second-order kinetic model [37].

The pseudo-first-order kinetic model assumes that the uptake rate of Fe(II) and Mn(II) with time is directly proportional to the amount of available active sites on the adsorbent surface. The pseudo-first-order kinetic model equation is given as

(3) $$\begin{array}{c} \ln\left(q_{e}-q_{t}\right)=\ln q_{e}-k_{1}t, \end{array}$$

where *q*_*e*_ and *q*_*t*_ are the uptake amount (mg/g) at equilibrium and *t* (time), respectively, and *k*_1_ is the pseudo-first-order adsorption rate constant (min⁡^−1^). The biosorption rate constants (*k*_1_) can be determined experimentally by plotting of ln⁡(*q*_*e*_ − *q*_*t*_) against *t*.

The pseudo-second-order kinetic model assumes that chemical adsorption can be the rate limiting stage involving valence forces through sharing or exchange of electrons between adsorbent and adsorbate. The pseudo-second-order kinetic equation is expressed as

(4) $$\begin{array}{c} \frac{t}{q_{t}}=\frac{1}{K_{2}q_{e}^{2}}+\frac{t}{q_{e}}, \end{array}$$

where *k*_2_ (g/mg min) is the rate constant of the second-order equation, *q*_*t*_ (mg/g) is the amount of biosorption time *t* (min), and *q*_*e*_ is the amount of biosorption equilibrium (mg/g).

**Figure 6:**
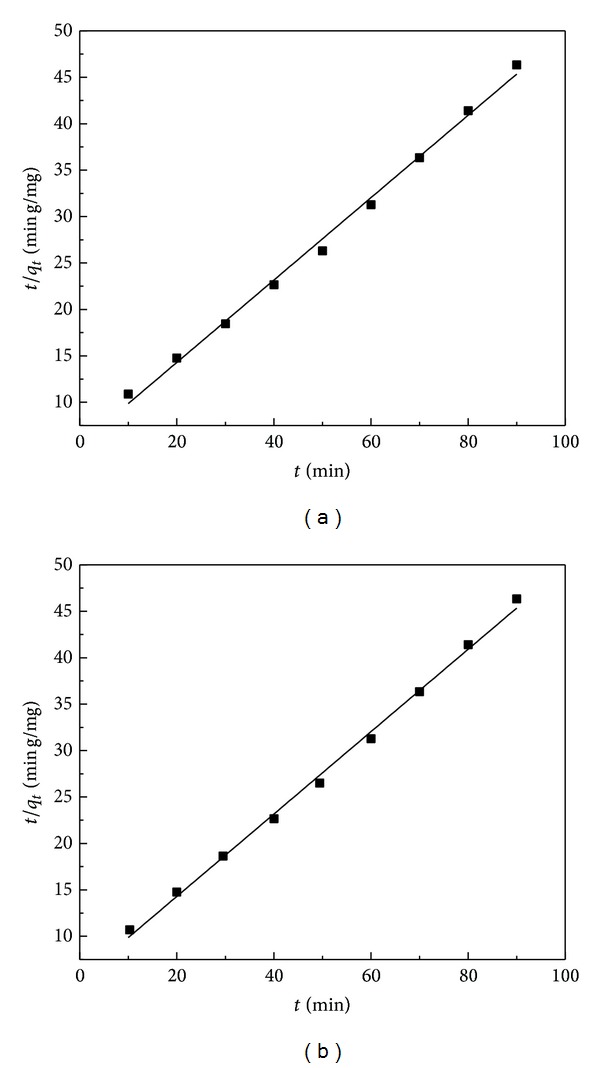
Pseudo-second-order kinetic plots onto RHA: (a) for Fe(II) biosorption and (b) for Mn(II) biosorption (metal concentration: 20 mg/L; adsorbent dosage: 0.6 g/100 mL; contact time: 90 min; pH: 5 and 6, resp.).

Results show that the pseudo-second-order model was more appropriate for the adsorption of Fe(II) and Mn(II). The correlation coefficients of adsorption using RHA for pseudo-second-order kinetic model are both closer to unity than those for the pseudo-first-order kinetic model. The linear plots of *t*/*q*_*t*_ against *t* for the pseudo-second-order model for the biosorption of Fe(II) and Mn(II) ions onto RHA are shown in Figures 6(a) and 6(b), respectively. The rate constants (*k*_2_) and the *R* ^2^ and the *q*_*e*_ values are given in Table 2. And the theoretical *q*_*e*_ values of the RHA agree well with the experimental *q*_*e*_ values compared with those for the pseudo-first-order kinetic model. While the theoretical *q*_*e*_ values of the rice husk adsorption for the two kinetic models both agree well with the experimental *q*_*e*_ values, the kinetic models fit well with the adsorption process and confirm the chemisorption of Fe(II) and Mn(II) onto rice husk.

### 3.7. Adsorption Isotherm Models

Adsorption isotherms describe the adsorption process and how adsorbates interact with a biosorbent. It is important to establish the most acceptable correlations for the batch equilibrium data for analysis and design of adsorption systems. The most frequently used models to describe the equilibrium data of adsorption are Langmuir, Freundlich, and Dubinin-Radushkevich isotherm models. In the present work, the three models were applied in the study of adsorption isotherms of Fe(II) and Mn(II).

The Langmuir model assumes that the uptake of metal ions is monolayer sorption on a homogenous surface and without any interaction between adsorbed ions [38]. This model is represented by the following equation:

(5) $$\begin{array}{c} \frac{C_{e}}{q_{e}}=\frac{C_{e}}{q_{m}}+\frac{1}{K_{L}q_{m}}, \end{array}$$

where *C*_*e*_ is the equilibrium concentration of Fe(II) and Mn(II) in solutions (mg/L), *q*_*e*_ is the equilibrium concentration of Fe(II) and Mn(II) on the biosorbent (mg/g), *q*_*m*_ is the monolayer biosorption capacity of the biosorbent (mg/g), and *K*_*L*_ is the Langmuir biosorption constant (L/mg).

Feasibility of the Langmuir isotherm in terms of a dimensionless constant was expressed by separation factor or equilibrium parameter, *R*_*L*_ [39]. The equation is expressed as follows:

(6) $$\begin{array}{c} R_{L}=\frac{1}{1+K_{L}C_{0}}. \end{array}$$

The Freundlich model assumes a heterogeneous adsorption surface and active sites with different energy [40]. Freundlich model is represented by the following equation:

(7) $$\begin{array}{c} q_{e}=K_{F}Ce^{1/n}. \end{array}$$

The linearized logarithmic form of the equation is

(8) $$\begin{array}{c} \log q_{e}=\log K_{F}+\frac{1}{n}\log C_{e}, \end{array}$$

where *K*_*F*_ is the Freundlich constant of the relative adsorption capacity of the adsorbent and the empirical parameter 1/*n* indicates the adsorption intensity.

Smaller value of 1/*n* implies stronger interaction between the adsorbent and heavy metal, while 1/*n* values exist between 0 and 1 indicating the identical adsorption process and adsorption energies for all sites [41].

**Figure 7:**
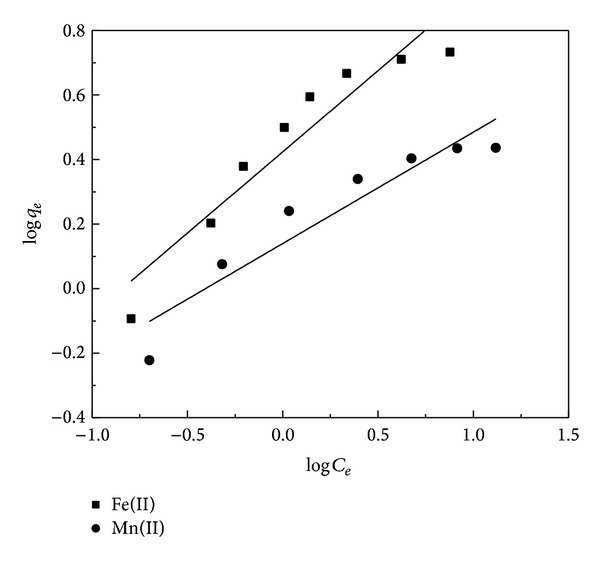
Freundlich isotherm plots for the biosorption of Fe(II) and Mn(II) onto RHA (adsorbent dosage: 0.6 g/100 mL; contact time: 90 min; pH: 5 and 6, resp.).

**Figure 8:**
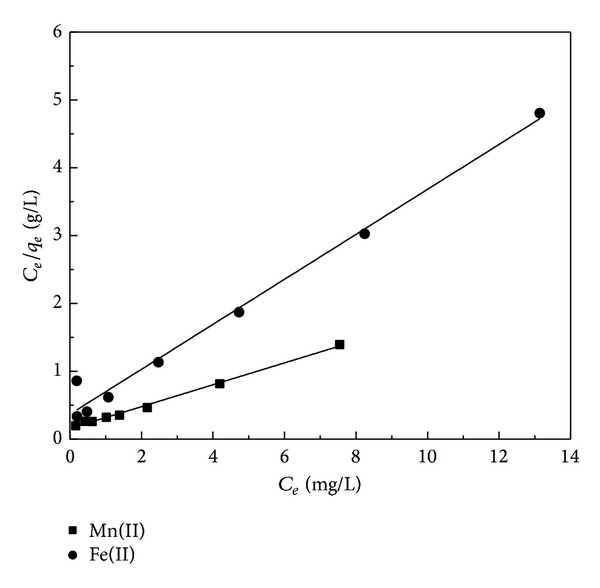
Langmuir isotherm plots for the biosorption of Fe(II) and Mn(II) onto RHA (adsorbent dosage: 0.6 g/100 mL; contact time: 90 min; pH: 5 and 6, resp.).

Figure 7 shows the nonlinear Freundlich isotherms which were obtained by plotting log⁡*q*_*e*_ against log⁡*C*_*e*_ values. From these plots the values of the *R* ^2^ were found to be 0.901 for Fe(II) biosorption and 0.892 for Mn(II) biosorption. However, the Langmuir isotherm was obtained by plotting *C*_*e*_/*q*_*e*_ against *C*_*e*_ values, and the correlation coefficients (*R* ^2^) were 0.995 and 0.986 for Fe(II) and Mn(II), respectively (Figure 8). These results indicate that the Freundlich model was not adequate to describe the relationship between the amounts of adsorbed metal ions and their equilibrium concentration in the solution. Therefore, the result showed that the Langmuir isotherm model fitted well with the equilibrium data as it presents higher *R* ^2^ values. The parameters of Fe(II) and Mn(II) adsorption isotherms for RHA are shown in Table 3. For favorable adsorption the value of *R*_*L*_ should range in between 0 and 1. The *R*_*L*_ values for the adsorption process were estimated at initial concentration from 5 to 40 mg/L of Fe(II) and Mn(II) ions. For all the experimental data, these values are lying between 0 and 1 and indicated favorable adsorption [42]. The *R*_*L*_ values of Fe(II) and Mn(II) in Langmuir isotherm are shown in Table 4.

Sorption energy was calculated by the D-R isotherm model to determine the nature of biosorption processes as physical or chemical. The linear presentation of the D-R isotherm equation [43] is expressed as follows:

(9) $$\begin{array}{c} \ln q_{e}=\ln q_{m}-\beta \varepsilon^{2}, \end{array}$$

where *q*_*e*_ is the amount of metal ions adsorbed on per unit weight of biomass (mol/L), *q*_*m*_ is the maximum biosorption capacity (mol/g), *β* is the activity coefficient related to biosorption mean free energy (mol^2^/J^2^), *ε* is the Polanyi potential [44], and the equation is given as

(10) $$\begin{array}{c} \varepsilon =\text{RT}\;\ln\left(1+\frac{1}{C_{e}}\right). \end{array}$$

The biosorption mean free energy (*E*; kJ/mol) is described as

(11) $$\begin{array}{c} E=\frac{1}{\sqrt{-2\beta }}. \end{array}$$

The biosorption mean free energy gives information about biosorption mechanism. If *E* value is between 8 and 16 kJ/mol, the biosorption process was chemical ion exchange in nature and if *E* < 8 kJ/mol, it was physical in nature [45, 46]. The mean biosorption energy was calculated as 2.53 and 2.27 kJ/mol for the biosorption of Fe(II) and Mn(II) ions, respectively (Table 3). These results suggest that the biosorption processes of both metal ions onto RHA were physical in nature because the sorption energies were less than 8 kJ/mol.

### 3.8. Comparison Study

Table 5 gives the maximum capacities of different adsorbents for the removal of Fe(II) and Mn(II) from aqueous solutions. It can be seen that maximum adsorptive capacities for these metal ions were different for different materials used. This will depend on the physical nature and chemical composition of the materials used from removal of metal ions. It can be seen from the table that rice husk ash has comparable adsorption capacity with respect to other adsorbents reported in the literature [47].

## 4. Conclusions

This study focused on the biosorption of Fe(II) and Mn(II) ions onto RHA from aqueous solution. The operating parameters, pH of the solution, contact time, adsorbent dosage, and initial concentration, were effective on the biosorption efficiency of Fe(II) and Mn(II). The maximum biosorption capacity (*q*_*m*_) of RHA was found to be 6.211 mg/g for Fe(II) ions and 3.016 mg/g for Mn(II) ions. The equilibrium adsorption experiments fitted well with Langmuir than Freundlich isotherm models and showed a correlation coefficient *R* ^2^ equals 0.995 and 0.986, respectively. The mean free energy values evaluated from the D-R model indicated that the biosorption of Fe(II) and Mn(II) onto RHA has taken place by physical sorption in nature. The equilibrium data indicated that the biosorption of Fe(II) and Mn(II) ions onto RHA followed well the pseudo-second-order kinetic model. It can also be concluded that the RHA is an effective and alternative material for the removal of Fe(II) and Mn(II) ions from wastewater because of its high biosorption capacity, cost-effectiveness, and abundant availability.

## Figures and Tables

**Table 1:** XRF analysis on the RHA before and after adsorption of Fe(II) and Mn(II) (%).

Element	Unloaded biomass	Fe(II)-loaded biomass	Mn(II)-loaded biomass
O	26.650	24.530	24.183
Na	0.040	—	—
Mg	0.301	0.090	0.057
Al	0.132	0.014	0.012
Si	29.433	27.538	27.276
P	0.511	0.054	0.054
S	0.102	0.154	0.069
Cl	0.190	0.342	0.097
K	2.123	0.338	0.290
Ca	0.942	0.061	0.048
Mn	0.183	0.187	0.293
Zn	0.011	0.002	0.003
Fe	—	0.175	0.013
Cu	—	0.003	0.004

**Table 2:** Kinetic parameters obtained from pseudo-first-order and pseudo-second-order for the Fe(II) and Mn(II) adsorption onto RHA.

	*q*_e,exp⁡_ (mg/g)	Pseudo-first-order			Pseudo-second-order		
		*K*_1_ (min^−1^)	*q*_e,calc_ (mg/g)	*R* ^2^	*K*_2_ (g/mg·min)	*q*_e,calc_ (mg/g)	*R* ^2^
Fe(II)	1.943	0.0714	2.2182	0.981	0.0361	2.2573	0.995
Mn(II)	1.936	0.0691	2.4717	0.974	0.0344	2.262	0.997

**Table 3:** Isotherm parameters for the adsorption of Fe(II) and Mn(II) on the RHA.

	Freundlich isotherm model			Langmuir isotherm model			D-R isotherm model		
	*K*_*F*_	1/*n*	*R* ^2^	*K*_*L*_ (L/mg)	*q*_*m*_ (mg/g)	*R* ^2^	*E* (kJ/mol)	*q*_*m*_ (mg/g)	*R* ^2^
Fe(II)	2.649	0.503	0.901	1.032	6.211	0.995	2.53	4.49	0.917
Mn(II)	1.379	0.345	0.892	0.907	3.016	0.986	2.27	2.54	0.918

**Table 4:** The *R*_*L*_ value of Fe(II) and Mn(II) in Langmuir isotherm.

Different initial concentration (mg/L)	2	5	10	20	25	30	35	40
*R*_*L*_ value of Fe(II)	0.3264	0.1623	0.0883	0.0462	0.0373	0.0313	0.0269	0.0237
*R*_*L*_ value of Mn(II)	0.3554	0.1807	0.0993	0.0522	0.0422	0.0354	0.0305	0.0268

**Table 5:** Comparison of maximum adsorption capacities of different adsorbents for Fe(II) and Mn(II) ions.

	Serial no.	Adsorbents	Adsorption capacity (mg/g)	Contact time (min)	Concentration range (mg/L)	pH	Temp. range (K)	References
Fe(II)	1	Coir fibres	2.84	120	73.50–83.9	5	308	[48]
	2	Modified coir fibres	7.49	120	73.50–83.9	5	308	[48]
	3	Activated carbon from coconut shells	81.89	90	20–100	6	298	[10]
	4	Chitosan/polyethylene glycol blend membrane	90.9	80	2–10	5	300	[49]
	5	Pine bark wastes	2.03	30	55.6–111.2	4	303–333	[50]
	6	Chitosan	57.5	40	3–9	5	—	[51]
	7	Cross-linked chitosan	64.1	60	3–9	5	—	[51]
	8	Rice husk ash	6.21	60	2–40	5	298	Present study

Mn(II)	9	*Pithecellobium dulce* carbon	7.0	50	5–25	7	—	[34]
	10	Crab shell particles	69.9	120	10–1000	6	296	[52]
	11	*Bombax malabaricum *	8.2	50	5–25	7	—	[35]
	12	*Pithecellobium dulce *	7.0	90	5–25	9	—	[35]
	13	*Ipomea batatas *	6.0	90	5–25	9	—	[35]
	14	*Peltophorum ferrugineum *	5.5	90	5–25	7	—	[35]
	15	Activated zeolite with NaCl	0.78	150	5–600	6	298	[30]
	16	Manganese oxide coated zeolite	1.1	150	25–600	6	298	[53]
	17	Chitosan/polyethylene glycol blend membrane	21.7	80	2–10	6	300	[48]
	18	Activated carbon from coconut shells	75.65	90	20–100	7	298	[10]
	19	Tannic acid immobilised activated carbon	1.13	60	1–10	7	298	[54]
	20	Rice husk ash	3.02	60	2–40	6	298	Present study
